# *Mycobacterium avium* Subspecies *paratuberculosis* and Bovine Leukemia Virus Seroprevalence and Associated Risk Factors in Commercial Dairy and Beef Cattle in Northern and Northeastern China

**DOI:** 10.1155/2015/315173

**Published:** 2015-10-04

**Authors:** Wu-Wen Sun, Wen-Fa Lv, Wei Cong, Qing-Feng Meng, Chun-Feng Wang, Xiao-Feng Shan, Ai-Dong Qian

**Affiliations:** ^1^College of Animal Science and Technology, Jilin Agriculture University, Changchun, Jilin 130118, China; ^2^Jilin Entry-Exit Inspection and Quarantine Bureau, Changchun, Jilin 130000, China

## Abstract

*Mycobacterium avium* subspecies *paratuberculosis* (MAP) and bovine leukemia virus (BLV) are important pathogens, commonly responsible for economical loss to cattle farms all over the world, yet their epidemiology in commercial dairy and beef cattle in China is still unknown. Thus, from September 2013 to December 2014, a large-scale seroprevalence study was conducted to determine the seroprevalence and identify herd-level risk factors associated with MAP and BLV infection. The source sample was 3674 cattle from 113 herds in northern and northeastern China. Antibodies against MAP and BLV were detected using ELISA tests. At animal-level, the seroprevalence of antibodies against MAP and BLV was 11.79% (433/3674) and 18.29% (672/3674), respectively. At herd-level, the seroprevalence of antibodies against MAP and BLV was 20.35% and 21.24% (24/113), respectively. Herd size was identified to be associated with MAP infection while herd size and presence of cattle introduced from other farms were significantly associated with BLV infection. Further research is needed to confirm these findings and improve the knowledge of the epidemiology of these two pathogens in these regions and elsewhere in China.

## 1. Introduction

Paratuberculosis, also known as Johne's disease caused by* Mycobacterium avium* subsp.* paratuberculosis* (MAP), is a chronic infectious granulomatous enteritis of global importance in primarily domestic and wild ruminants [1, 2]. This infection can induce significant economic losses in cattle due to reduced milk production and premature culling [3], decreased value at slaughter [4], and eventual death [5]. Other economic losses can also be caused by the presence of paratuberculosis in cattle herds including reduced feed efficiency, decreased milk's fat and protein content, decreased fertility, and increased incidence of mastitis [6].

Bovine leukemia virus (BLV), an exogenous C-type oncovirus retrovirus, is the causative pathogen of enzootic bovine leucosis (EBL), lymphomas in bovine species [7]. Most of infected cattle do not develop clinical symptoms. However, nearly one-third of BLV carriers develop a form of the disease known as persistent lymphocytosis (PL), and malignant B-cell lymphosarcomas can only be found in 1–5% of BLV-infected animals [7]. BLV infection has a global distribution, and EBL is an important group of commonly major diseases that have huge implications in economic losses around the world and cause a sanitary barrier to international trade [8]. In some countries, EBL has been successfully uprooted via national control and elimination programs, such as those used in Europe [9, 10].

To date, very limited information on the seroprevalence of MAP and BLV infections in cattle is available in China. In order to address this lack of publication issue, the objective of this study was to describe the seroprevalence of MAP and BLV infections in commercial dairy and beef cattle in northern and northeastern China and clarify risk factors associated with seroprevalence of MAP and BLV in the study regions.

## 2. Materials and Method

### 2.1. Ethics Statement

This study was approved by the Animal Ethics Committee of Jilin Agriculture University, China. Cattle of various categories (dairy cattle and beef cattle), from which serum samples were collected, were handled with good ethical animal practices required by the Animal Ethics Procedures and Guidelines of the People's Republic of China. The collection of serum samples was performed as part of routine process of disease monitoring and surveillance program for these cattle. The owners of cattle had given permission for collecting the serum samples.

### 2.2. Serum Samples

**Figure 1:**
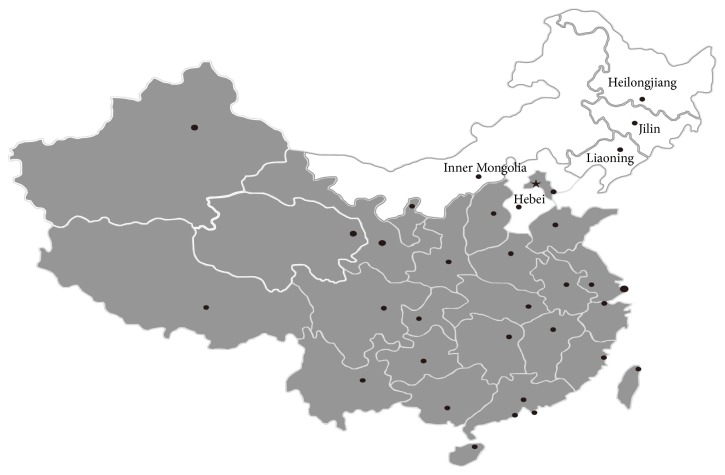
Geographic distribution of cattle sampled regions in China.

A total of 3674 bovine blood samples were collected from four provinces (Heilongjiang, Jilin, Liaoning, Hebei, and Inner Mongolia Autonomous Region) (Figure 1) between September 2013 and December 2014. Blood samples were collected from 12 administrative districts and 113 herds with 50 cattle and more across the five regions. Cattle sample sources from each farm were selected randomly using a table of random digits. Several large-scale farms (with more than 150 livestock animals) were not included because the owner was often not present to give permission. In the case of cattle, approximately 10% in each farm were sampled. All of the animals sampled were clinically healthy. Blood samples (10 mL) of each animal were collected by puncturing the tail vessel using sterile tubes sans/without anticoagulant. Samples were kept at 4°C and centrifuged at 1000 ×g for 15 min. The serum was separated and stored at −20°C until analysis.

### 2.3. Serological Examination

The serological examination of MAP was performed by the commercial antibody detection kits (Pourquier-IDEXX ELISA Paratuberculosis Screening and Verification Ab Test) following the recommended protocol. The antibodies to BLV were detected using a commercially available verification ELISA kit (Pourquier, Montpellier, France) following the manufacturer's instructions.

### 2.4. Epidemiological Data Collection

Data obtained with the epidemiological questionnaires were used in the analysis of risk factors associated with the animal-level and herd-level target prevalence. For animal-level variables, information about species, geographic origin (Heilongjiang/Jilin/Liaoning/Hebei/Inner Mongolia Autonomous Region), age, gender, and abortion history was acquired from farm administrators. For herd-level variables, the respective categories were as follows: region (Heilongjiang/Jilin/Liaoning/Hebei/Inner Mongolia Autonomous Region), herd size (small (50–100)/medium (100–150)/large (>150)), mixed farming (yes: dairy and beef cattle/no: dairy cattle only or beef cattle only), methods of cattle house cleaning (not practiced/sweeping/water hosing), source of water (well/tap water), management system (intensive: very strict control for irrelevant personnel and animals/semi-intensive: slight control for irrelevant personnel and animals/extensive: no control for irrelevant personnel and animals), presence of cattle introduced from other farms (yes/no), presence of other animals (yes/no), type of production (beef/milk/mixed), and veterinary service (yes/no).

### 2.5. Statistical Analysis

In this study, an infected farm was defined as a farm with one or more infected animals. Explanatory variables thought to influence the MAP and BLV seroprevalence were in two broad categories: animal-level variables and herd-level variables. Explanatory variables were tested for their relationship with the MAP and BLV seroprevalence by the Mantel-Haenszel Chi-square test and bivariate analyses. Variables were included in the multivariate analysis if they had a *P* value of equal to or less than 0.20 in the bivariate analysis. Region was used as a concomitant variable in the multivariate analysis. Adjusted odds ratio (OR) and its 95% confidence interval were calculated by multivariate logistic regression analysis. A *P* value less than 0.05 was considered statistically significant. Results were analyzed with SPSS 19.0 software package.

## 3. Results

### 3.1. Seroprevalence and Risk Factor Analysis of MAP

At animal-level, of the 3674 samples tested, positive results for anti-MAP antibodies were observed in 433 samples, representing a prevalence of 11.79% (95% confidence interval (CI): 15.65–19.64). The results of bivariate analyses are shown in Table 1. Of these, the seroprevalence of MAP was diverse in different regions; the most frequent level was 14.12% (95/673) in Liaoning province, followed by 13.48% (86/638) in Jilin province, 12.12% (72/594) in Heilongjiang province, 11.18% (107/957) in Inner Mongolia Autonomous Region, and 10.25% (73/712) in Hebei province. Among different ages of cattle, the highest seroprevalence of MAP infection was seen in cattle aged ≥6 years (18.40%). With respect to gender, the seroprevalence in male cattle (12.84%) was higher than that in female cattle (11.22%). Moreover, cattle with abortion history (20.70%) have a significantly higher seroprevalence than that in cattle without abortion history (10.34%) (*P* < 0.001).

The herd-level prevalence was 20.35% (23/113, 95% CI: 12.93–27.78). The results of the bivariate analysis for risk factors are presented in Table 2. The herd-level prevalence was 10.53% in Heilongjiang province, 16.67% in Jilin province, 28.57% in Liaoning province, 22.22% in Hebei province, and 22.73% in Inner Mongolia Autonomous Region. The variables selected (*P* ≤ 0.2) for the multivariate analysis were as follows: herd size, methods of cleaning, source of water, and presence of cattle introduced from other farms. Herd size was identified to be associated with MAP infection in cattle in the multivariate analysis (Table 4).

### 3.2. Seroprevalence and Risk Factor Analysis of BLV

Overall, at animal-level sample source, 672 out of 3674 serum samples (18.29%, 95% CI: 48.64–51.56) were seropositive for BLV by ELISA test. The results of bivariate analyses are shown in Table 1. Among different types of cattle, dairy cattle (18.49%) had a little higher seroprevalence than that in beef cattle (18.04%). In terms of geographical origin, BLV seroprevalence varied in cattle from different regions, ranging from 16.61% in Inner Mongolia Autonomous Region to 21.00% in Jilin province. Positive cattle were found in all four age groups, varied from 12.86% to 24.72%, and the highest seroprevalence was detected in cattle of more than 6-year-old category. Seroprevalence of BLV in male and female cattle was 16.76% (214/1277) and 19.11% (458/2397), respectively. Cattle with abortion history (22.65%) have significantly higher seroprevalence than that in cattle without abortion history (17.58%) (*P* = 0.006).

The herd-level prevalence was 21.24% (24/113, 95% CI: 13.70–28.78). The results of the bivariate risk factors analysis are presented in Table 2. The herd-level prevalence was 21.05% in Heilongjiang province; 16.67% in Jilin province; 23.81% in Liaoning province; 25.93% in Hebei province; and 18.18% in Inner Mongolia Autonomous Region. The variables selected (*P* ≤ 0.2) for the multiple analysis were as follows: herd size, methods of cleaning, source of water, management system, presence of cattle introduced from other farms, type of production, and veterinary service. Herd size and presence of cattle introduced from other farms were significantly associated with BLV infection in the studied cattle in the multivariate analysis (Table 4).

## 4. Discussion

This is the first large-scale study of MAP and BLV antibody prevalence in commercial dairy cattle and beef cattle in China. High animal-level (11.79%; 95% CI: 7.3–15.4) and herd-level (20.35%; 95% CI: 30.2–39.1) MAP prevalence was found in northern and northeastern China, whereas high animal-level (18.29%; 95% CI: 7.3–15.4) and herd-level (21.24%; 95% CI: 30.2–39.1) BLV prevalence was also found in the same regions, indicating that the infections are widely spread in the study regions.

In the present study, animal-level MAP seroprevalence was 11.79%, but animal-level prevalence might even be higher. It is important to highlight that paratuberculosis has a comparatively long latent period, and the antibody levels against MAP only can be detected at the end of the latent period by the ELISA test, results to show that this method was noneffective for detecting infected animals in the early stages of the infection [11]. Thus, the animals that are infected at a young stage (i.e., no more than one year) will still exist in the herd, shedding MAP via feces and contaminating water and food, and will only present clinical symptoms in the adult stage [12]. However, paratuberculosis is an unknown disease for most farmers in the study regions, and although the high infection rates at animal-level and herd-level prevalence were found in these regions, most farmers are not aware of the impact of the infection and the economic losses that it can cause.

The seroprevalence of MAP significantly increased with cattle age (*P* < 0.001) (Table 3) and was also significantly higher in cattle herds with > 150 cattle compared with smaller herds (*P* = 0.024). An increase in seropositivity with herd size has also been reported in England [13] and Denmark [14]. This is not surprising because MAP is infective with an environmental component and density dependent effects are to be expected.

One of the critical issues is the probable zoonotic link between MAP and Crohn's disease, although this relation is still a vexed question and is the target of continual debates in science [15]. However, MAP has been detected in fresh and pasteurized milk [16–18], and the habit of consuming fresh and pasteurized milk is widespread in family farms in the study regions. Therefore, the validation of a zoonotic link between MAP and Crohn's disease could have great consequences for the cattle industry of the study regions and China.

BLV has a worldwide distribution and prevalence varies between countries [9, 10]. In the present study, this is the first report of BLV infection with a 18.29% seroprevalence at animal-level sample in cattle in China, which is lower than the 70% seroprevalence in Argentina [19], 41% in USA [20], and 32.5% in Japan [8]; however, the prevalence of BLV is much higher than the values of 2.28% in dairy cattle in Turkey [21]. Several factors may contribute to such differences, such as geographical conditions, the types and size of the cattle tested, and the specificity and sensitivity of the detection methods.

Analyses of risk factors for the animal-level sample presence of BLV revealed that age was a common risk factor for dairy and beef cattle. The seroprevalence of BLV infection in older cattle was significantly higher than that in younger cattle (Table 3). This is probably due to the fact that older cattle had more chance to contact this pathogen comparing to younger cattle, thus increasing the risk of infection.

Analyses of risk factors for the herd-level presence of BLV revealed that the herd size and presence of cattle introduced from other farms were a common risk factor for dairy and beef farms. Prevalence has an increasing trend with increasing herd size. The reason for this phenomenon is because the population of susceptible animals is greater in large herds. In addition, the most important risk factor for the introduction of not only exotic but also endemic diseases is movement of animals [22]. In China, farm owners rarely test animals for BLV infection ahead of their introduction into their own farms. Resultant, control measures such as negative confirmation of BLV in cattle before their introduction onto a farm should be conducted to prevent BLV infection.

In conclusion, this study firstly reported seroprevalence of MAP and BLV infection in commercial dairy and beef cattle in northern and northeastern China and also identified several herd-level risk factors associated with the presence of seropositive cattle. Future research is required to compare the influence of each factor responsible for these pathogens infection at animal- and herd-level in these regions and elsewhere in China.

## Figures and Tables

**Table 1:** Bivariate analysis for the animal-level seropositivity of *Mycobacterium avium *subspecies* paratuberculosis* (MAP) and bovine leukemia virus (BLV) infection in commercial dairy and beef cattle in northern and northeastern China.

Characteristics	Cattle tested	MAP			BLV		
	(number)	Number of positive cattle	%	*P* value	Number of positive cattle	%	*P* value
Species							
Dairy cattle	2044	251	12.28	0.317	378	18.49	0.722
Beef cattle	1630	182	11.17		294	18.04	
Region							
Heilongjiang	594	72	12.12	0.150	103	17.34	0.126
Jilin	638	86	13.48		134	21.00	
Liaoning	673	95	14.12		131	19.47	
Hebei	712	73	10.25		145	20.37	
Inner Mongolia Autonomous Region	957	107	11.18		159	16.61	
Age							
≤1 year	995	98	9.85	<0.001	128	12.86	<0.001
2-3 year	1278	103	8.06		226	17.68	
4-5 year	689	101	14.66		142	20.61	
≥6 year	712	131	18.40		176	24.72	
Gender							
Male	1277	164	12.84	0.147	214	16.76	0.079
Female	2397	269	11.22		458	19.11	
Abortion history							
No	3162	327	10.34	<0.001^*∗*^	556	17.58	0.006
Yes	512	106	20.70		116	22.65	
Total	3674	433	11.79		672	18.29	

**Table 2:** Bivariate analysis for the herd-level seropositivity of *Mycobacterium avium *subspecies* paratuberculosis* (MAP) and bovine leukemia virus (BLV) infection in commercial dairy and beef farms in northern and northeastern China.

Variables	Number of the sampled	MAP		BLV	
		Number of positive cattle (%)	*P*	Number of positive cattle (%)	*P*
Region					
Heilongjiang	19	2 (10.53)	0.673	4 (21.05)	0.930
Jilin	24	4 (16.67)		4 (16.67)	
Liaoning	21	6 (28.57)		5 (23.81)	
Hebei	27	6 (22.22)		7 (25.93)	
Inner Mongolia Autonomous Region	22	5 (22.73)		4 (18.18)	
Herd size					
Small (50–100)	52	9 (17.31)	0.024	8 (15.38)	0.002
Medium (100–150)	46	7 (15.22)		6 (13.04)	
Large (>150)	15	7 (46.67)		8 (53.33)	
Mixed farming					
Yes	33	7 (21.21)	0.884	8 (24.24)	0.616
No	80	16 (20.00)		16 (20.00)	
Methods of cleaning					
Not practiced	13	6 (46.15)	0.038	7 (53.85)	0.007
Sweeping	39	8 (20.51)		8 (20.51)	
Water hosing	61	9 (14.75)		9 (14.75)	
Source of water					
Well	41	11 (26.83)	0.197	13 (31.70)	0.040
Tap water	72	12 (16.67)		11 (15.28)	
Management system					
Intensive	35	4 (11.43)	0.227	3 (8.57)	0.036
Semi-intensive	46	10 (21.74)		10 (21.74)	
Extensive	32	9 (28.13)		11 (34.38)	
Presence of other animals					
No	76	15 (19.74)	0.815	16 (21.05)	0.945
Yes	37	8 (21.62)		8 (21.62)	
Presence of cattle introduced from other farms					
No	81	14 (17.28)	0.197	13 (16.05)	0.032
Yes	32	9 (28.13)		11 (34.38)	
Type of production					
Beef	26	3 (11.54)	0.245	5 (19.23)	0.161
Milk	72	15 (20.83)		13 (18.06)	
Mixed	15	5 (33.33)		6 (40.00)	
Veterinary service					
No	43	11 (25.58)	0.279	14 (32.56)	0.021
Yes	70	12 (17.14)		10 (14.29)	

**Table 3:** Multivariate analysis of risk factors associated with animal-level prevalence of *Mycobacterium avium *subspecies* paratuberculosis* (MAP) and bovine leukemia virus (BLV) in commercial dairy and beef cattle in northern and northeastern China.

Agents	Variables^a^	Adjusted odds ratio^b^	95% confidence interval	*P* value
MAP	Age			
	≤1 year	Reference		
	2-3 year	0.792	0.594–1.056	0.112
	4-5 year	1.503	1.117–2.020	0.007
	≥6 year	2.033	1.537–2.689	<0.001
	Abortion history			
	No	Reference		
	Yes	2.334	1.833–2.971	<0.001

BLV	Age			
	≤1 year	Reference		
	2-3 year	1.511	1.194–1.913	<0.001
	4-5 year	1.775	1.363–2.311	<0.001
	≥6 year	2.337	1.816–3.009	<0.001

^a^The variables included were those with a *P* ≤ 0.20 obtained in the bivariate analysis.

^b^Adjusted by region and the rest of characteristics included in this table.

**Table 4:** Multivariate risk factors analysis associated with herd-level prevalence of *Mycobacterium avium *subspecies* paratuberculosis* (MAP) and bovine leukemia virus (BLV) in commercial dairy and beef farms in northern and northeastern China.

Agents	Variables^a^	Adjusted odds ratio^b^	95% confidence interval	*P* value
MAP	Herd size			
	Small (50–100)	Reference		
	Medium (100–150)	0.858	0.292–2.522	0.780
	Large (>150)	4.181	1.206–14.490	0.019

BLV	Herd size			
	Small (50–100)	Reference		
	Medium (100–150)	0.825	0.263–2.584	0.741
	Large (>150)	6.286	1.777–22.240	0.002
	Presence of cattle introduced from other farms			
	No	Reference		
	Yes	2.740	1.070–7.016	0.041

^a^The variables included were those with a *P* ≤ 0.20 obtained in the bivariate analysis.

^b^Adjusted by region and the rest of characteristics included in this table.
